# Supplementary material for: Crawling and Gliding: A Computational Model for Shape-Driven Cell Migration
Source: PLoS Comput Biol. 2015 Oct 21;11(10):e1004280. doi: 10.1371/journal.pcbi.1004280 (PMC4619082; doi:10.1371/journal.pcbi.1004280)
Supplement: S1 Code — (ZIP) [file pcbi.1004280.s012.zip › release/tst/doc/html/functions_c.html]

Tissue Simulation Toolkit: Class Members


Tissue Simulation Toolkit
 0.1.4.1


- Main Page
- Namespaces
- Classes
- Files

- Class List
- Class Hierarchy
- Class Members

- All
- Functions
- Variables
- Related Functions

- a
- b
- c
- d
- e
- f
- g
- i
- j
- l
- m
- n
- o
- p
- q
- r
- s
- t
- v
- w
- x
- y
- z
- ~

Here is a list of all class members with links to the classes they belong to:

### - c -

- capacity
  : Cell
- Cell()
  : Cell
- cell
  : Dish
- CellBirth()
  : Cell
- CellDensity()
  : CellularPotts
- CellGrowthAndDivision()
  : Dish
- CellularPotts
  : Cell
  , CellularPotts
  , Dir
- ChangeTitle()
  : X11Graphics
- chem
  : Cell
- chemotaxis
  : Parameter
- CleanUp()
  : Parameter
- ClearGrad()
  : Cell
- ClearGrads()
  : Dish
- ClearImage()
  : QtGraphics
  , X11Graphics
- ClearJ()
  : Cell
- ClickCell()
  : Info
- colour
  : Cell
- Colour()
  : Cell
- colour\_of\_birth
  : Cell
- ColourOfBirth()
  : Cell
- Compactness()
  : CellularPotts
- conn\_diss
  : Parameter
- ConstructInitCells()
  : CellularPotts
- ConstructorBody()
  : Dish
- ContourPlot()
  : PDE
- CountCells()
  : Dish
- CPM
  : Dish
- CropSize()
  : X11Graphics


---

Generated on Thu Aug 14 2014 22:04:01 for Tissue Simulation Toolkit by  

 1.8.6
